# Dieting, body weight concerns and health: trends and associations in Swedish schoolchildren

**DOI:** 10.1186/s12889-020-8295-7

**Published:** 2020-02-05

**Authors:** Christina Berg, Christel Larsson

**Affiliations:** Department of Food and Nutrition, and Sport Science, University of Gothenburg, Box 300, 405 30 Göteborg, Sweden

**Keywords:** Dieting, Body weight concerns, Body image, Body dissatisfaction, Overweight, Adolescents

## Abstract

**Background:**

Dieting is a risk factor of both eating disorders and obesity. The aim was to examine time trends of dieting in Swedish adolescents, and explore how dieting and body weight dissatisfaction are related to self-reported health, wellbeing and health behaviours.

**Methods:**

Analyses of cross-sectional Swedish data from HBSC (Health Behaviour in School-aged Children) surveys 1994–2014. In total, about 30,000 girls and boys in the age of 11, 13 and 15 years participated. Data was collected by using classroom administered questionnaires in 5th, 7th and 9th grade. Logistic regressions was used to analyse secular trends of dieting, and how dieting and body dissatisfaction were associated with self-reported overall health, health behaviours, BMI and various physical, psychological and social aspects of health in 2014.

**Results:**

Dieting increased from 1994 to 2014 in both girls and boys in all age groups, and in 2014, the prevalence was 14% in girls and 8% in boys. The prevalence of body satisfaction was 65% respectively 69%. Body weight dissatisfaction and dieting were present in all body weight classes and were associated with self-reported poor health and many other negative health aspects. In comparison with the participants that were satisfied with their body weight the odds ratio (95% CI) for self-reported poor health was 3.4 (2.6–4.4) in dieters, 4.9 (3.8–6.4) in participants who perceived a need to lose weight and 2.1 (1.5–2.8) in those who perceived a need to gain weight, when adjusting for age, sex and body weight class.

**Conclusions:**

When promoting health among school age children body weight dissatisfaction and dieting ought to be considered. Furthermore, it is important to support girls and boys in all weight classes to reach and maintain a healthy body image and weight.

## Background

Young people of today face a strenuous life in an obesogenic society with abundance of attractive sedentary activities and choices of energy dense foods offered. At the same time they are exposed to an unrealistic thin fit body and a healthy lifestyle as ideals.

Weight reduction might be a way to prevent illness and diseases, though it might become an unhealthy action in a vicious circle. While the risk of future dieting and extreme weight loss practices tend to be higher among people with higher BMI [1–3], dieting in turn predicts obesity later in life [4, 5]. Likewise, although weight reduction might boost self-esteem [6] it is probable that unsuccessful dieting results in lower self-esteem. Young people with low self-esteem have in turn been shown to be more likely to make attempts to reduce weight [7, 8]. In addition to, or because of, the risk of ending up in a vicious circle due to these bidirectional associations, dieting and disordered eating in early ages are also likely to continue into adulthood [9]. Moreover, adolescent dieting might have negative impact of psychological as well as nutritional status [10]. Body dissatisfaction and early dieting might also result in other unhealthy behaviours with intention to reduce weight, e.g. smoking and purging [2, 11], and it is also a risk factor for eating disorders [12].

The perception of overweight and weight concerns rather than actual body weight class seems to initiate dieting [13, 14]. Misperception of overweight is common especially among girls. Data from 33 countries showed that a third of the 15-years old girls with normal or low weight perceived them self as overweight. In the Swedish sample, an increasing proportion of girls and boys regarded their body to be too fat. In 2014, the prevalence of feeling too fat was 47% respectively 22% among girls and boys. This is worrying since a clear association between body dissatisfaction and psychosomatic complaints was observed in both sexes [15]. Furthermore, another study including Swedish adolescents showed body appreciation to be associated with psychological well-being and self-esteem in both genders even if boys had higher body appreciation than girls [16]. In other countries, body image has been found to associated with self-rated health [17], quality of life [18, 19], problem behaviours [20] and exposure to bullying [21].

While many studies have focused on body image less is known about dieting. Thus, we need to know more about health and wellbeing in dieting young people and to follow the prevalence of dieting. Therefore, it is of interest to study if dieting has increased, and how it is related to different dimensions of health and lifestyle. The aim of the present paper is to examine time trends of dieting in Swedish adolescents between years 1994 and 2014, and to explore how dieting and body weight dissatisfaction are related to self-reported health, wellbeing and health behaviours in 2014.

## Methods

The Health Behaviour in School-aged Children (HBSC) study collects cross-sectional questionnaire data on nationally representative samples of 11-, 13- and 15- years old schoolchildren in Europe and some other countries every fourth years [22]. The purpose is to study girls’ and boys’ health behaviours, health indicators and contextual variables. Data is collected by using classroom administered questionnaires. This paper presents data from the surveys in 1993–94, 1997–98, 2001–02, 2005–06, 2009–10 and 2014 in Sweden, collected according to the international research protocol. Samples were selected separately for each age group using a 2-stage clustered sampling procedure where school was the primary sampling unite and class the second. The likelihood for a school to be selected was proportional to the number of pupils in the particular school (Pareto-sampling). One class was selected in each school and all pupils in the selected class were invited to participate. The response rates are presented in Table 1. The analyses of secular trends were based on data from 30,480 adolescents (5th grade, *n* = 10,407; 7th grade, *n* = 9639; 9th grade, *n* = 9892 were included in the analyses), and the cross-sectional analyses are based on the survey 2014 with 7867 schoolchildren. Students from 386 of 491 eligible schools participated in 2014 and in total 69% of the invited schoolchildren (5th grade, *n* = 2689; 7th grade, *n* = 2338; 9th grade, *n* = 2840) completed the questionnaire.

**Table 1:** Response rates in the six surveys

Survey year	Number	Response rate (%)
1993/94	3584	85
1997/98	3802	90
2001/02	3926	87
2005/06	4421	85
2009/10	6880	88
2013/14	7867	69

### Dieting and body weight dissatisfaction

In the 2014 survey, dieting, body weight satisfaction and dissatisfaction was measured with the question “At present are you on a diet or doing something else to lose weight?” with the responses: “No, my weight is fine”; “No, but I should lose some weight”, “No, because I need to put on weight”, “Yes”.

Dieting in the surveys 1993/94 and 1997/98 was measured with a slightly different question: “Are you on a diet to lose weight?” with three response options (the same as the four above but excluding the third one).

### Perceived health, wellness and disease

Self-rated health, which is considered as a valid measure of adolescent well-being [23], was measured with the question “Would you say your health is …? ” and four response options (excellent, good, fair or poor). When analysing the data, the response alternative “faire” and “poor” was merged and regarded as a response of poor health.

Wellness was measured with the question “How healthy and well do you think you are?” followed by four response options (very well, fairly well, not very well). When analysing the data the two first response alternatives were merged and regarded as feeling well.

Chronical disease was measured with the question “Do you have a long-term illness, disability, or medical condition (like diabetes, eczema, allergy, or ADHD) that has been diagnosed by a doctor” with response option yes/no.

### Life satisfaction

Life satisfaction was investigated by a picture of a ladder [24] where the top of the ladder “10” implied the best possible life and the bottom “0” implied the worst possible life. The participants were asked to indicate where on the ladder she/he in general perceived to be at the moment. The perception of having a low life satisfaction was defined as response category 0–5, as suggested in the HBSC protocol.

### Subjective psychological and somatic health complaints

The frequency of the subjective symptoms, headache and stomach ache, were measured with a question of how often during the last 6 month the symptom had occurred with five responses ranging from rarely or never to, about every day. The psychological health complaints feeling low and sleeping difficulties were measured in the same format. These two complaints were considered frequent if reported daily.

### Social support in school and at home

Peer relationship and support were measured with two items. One was the statement “I can talk about my problems with my friends” with responses from “1” to “7” and the two polar labelled “Very strongly disagree” and Very strongly agree”. The perception of not being able to talk about problems with friends was defined as response category one to three. The other item was formulated “Other students accept me as I am” with five response categories from “Strongly agree” to “Strongly disagree”. The perception of not being accepted was defined as responding disagree or strongly disagree.

Exposure to bullying was measured with the question “How often have you been bullied at school in the last couple of months?” With the five responses ranging from “I have not been bullied at school in the last couple of months” to “Several times a week”. The response was dichotomized into not have been bullied or have been bullied at least once or twice during the last couple of months.

Not being able to talk about problems with the family and acceptance by teachers was measured and dichotomized according to the same principle as peer relationship and support above.

Family meals were measured with one question about breakfast and one about dinner: “How often do you have breakfast/dinner with your mother or father?” with six responses ranging from “Never” to “Every day”. The category “Never” was used as an indication of never eating breakfast/dinner together with parents.

### Health behaviours

The frequencies of consumption of fruit, vegetables, sweets, and sugar sweetened beverages were assessed with the question “How many times a week do you usually eat or drink …” with seven responses from “Never” to “Every day, several times a day”. One question was used to measure breakfast consumption at weekdays and weekends: How often do you usually eat breakfast (more than a glass of milk or fruit juice). There were six responses for weekdays, from “I never have breakfast during the week” to “5 days”. Consuming sweets and sugar sweetened beverages daily and not consuming vegetables, fruits and breakfast (weekdays) daily were considered as cut-off point for less healthy behaviours.

Exercise was measured with the question “Outside school hours, how often do you usually exercise in your free time so much that you get out of breath or sweat?” with seven response options. No weekly exercise was considered cut-off point for a less healthy behaviour.TV use in week days with the question “How many hours a day, in your free time, do you usually spend watching TV, videos (including you tubes or similar services), DVDs, and other entertainment on screen? with nine response options for weekdays separately. Watching TV 4 hours or more on weekdays was considered as a cut-off point for less healthy behaviour.

Being a smoker was defined as not answering “I do not smoke” to the question “How often do you smoke tobacco at present?” In a similar way consuming alcohol was defined as not responding “I never drink alcohol” to “How many drinks containing alcohol do you have on a typical day when you are drinking?”

Sleeping habits at weekdays was measured with the question “When do you usually go to bed if you have to go to school next morning?” with 11 response options. Bed time after 24 at weekdays was considered as a less healthy habit.

### Demographic variables

The questionnaire also included questions about the participants’ country of birth, who they were living together with, and their mother’s and father’s occupation. Living with both mother and father was considered as living in a nuclear family. The information on type of occupation was used to form three categories of socioeconomic status (SES) i.e. both parents, one parent respectively none of the parents having a non-manual occupation.

### Body weight class

Self-reported data (*n* = 6112) on height, weight, date of birth and sex was used to assess body weight status [25] after excluding values +/− 4 sd for height [26] and other extreme values. International Obesity Task Force gender- and age specific BMI cut-off points [25] were applied when categorizing the participants in four body weight classes: underweight, normal weight, pre-obesity and obesity.

### Analyses

IBM SPSS.25 was used for the analyses. Time trends in dieting were analysed using logistic regression models, stratified for gender and age group, with dieting as dependent variable and survey year as independent. Prevalence estimates with 95% confidence intervals were adjusted for the cluster sampling design and weighted to take biased school drop out into consideration by using SPSS procedures for complex samples.

In the cohort examined 2014, logistic regressions were used to analyse the association between background factors and dieting, and how body weight dissatisfaction and dieting was associated with different health aspects and health behaviours. These models were adjusted for age and sex. Participants reporting body weight satisfaction were considered as a reference group against which the three groups of body weight dissatisfaction and dieting were compared regarding the risk of less favourable health profile. In the models with the measures of overall health as dependent variables we also adjusted for body weight class in a second step, and added SES, place of birth and family structure in a third step. The number of included participants in different analyses varies due to missing values.

## Results

**Fig. 1:**
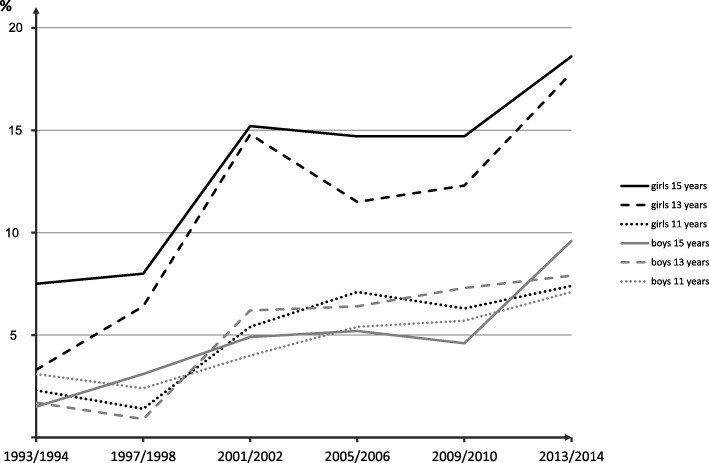
Secular trends in prevalence of dieting by age group and gender. Note that in the two first examinations the question about dieting was slightly differently formulated

The prevalence of dieting increased from 3% in 1994 to 11% in 2014, as illustrated in Fig. 1. This trend was significant for both girls and boys in all three age groups (*p* < 0.001). In 2014, 14% of the girls and 8% of the boys reported that they were trying to lose weight, and 14% of the girls and 10% of the boys that they did not but that they ought to do. Prevalence of dieting, body weight dissatisfaction and satisfaction in each grade are shown in Table 2.

**Table 2:** Prevalence of body weight satisfaction, dissatisfaction and dieting in Swedish schoolchildren participating in the national survey 2014

	All	Grade 5 (11 years)		Grade 7 (13 years)		Grade 9 (15 years)	
	% (95% CI)	Girls % (95% CI)	Boys % (95% CI)	Girls % (95% CI)	Boys % (95% CI)	Girls % (95% CI)	Boys % (95% CI)
Satisfied with weight	67 (66–68)	75 (73–78)	75 (72–78)	61 (57–64)	68 (64–72)	59 (55–62)	62 (59–65)
Need to gain weight	11 (10–12)	9 (7–11)	9 (8–11)	8 (6–10)	13 (11–16)	6 (5–8)	19 (17–21)
Need to lose weight	11 (10–12)	9 (7–11)	9 (7–11)	15 (13–18)	11 (9–13)	17 (15–19)	10 (8–12)
Trying to lose weight	11 (10–12)	7 (6–9)	7 (6–9)	17 (14–19)	8 (6–10)	18 (16–21)	9 (8–11)

As shown in Table 3, data from the survey in 2014 reveal that dieting or perceived need to lose weight was common also among participants without overweight. Furthermore, it is shown that the prevalence of dieting was higher in girls, among the older age groups, in non-nuclear families and in families with low SES (Table 4).

**Table 3:** Body weight satisfaction, dissatisfaction and dieting in relation to weight class of Swedish schoolchildren participating in the national survey 2014

	Under-weight (*n* = 720) % (95% CI)	Normal weight (*n* = 4428) % (95% CI)	Pre-obesity (*n* = 756) % (95% CI)	Obesity (*n* = 140) % (95% CI)
Satisfied with weight, %	61 (57–65)	74 (73–76)	40 (36–45)	11 (7–19)
Need to gain weight, %	35 (31–39)	9 (8–10)	2 (1–3)	3 (1–9)
Need to lose weight, %	1 (1–3)	8 (7–9)	34 (30–37)	44 (36–53)
Trying to lose weight, %	3 (2–5)	9 (8–10)	24 (21–28)	41 (33–49)

**Table 4:** Factors associated with dieting^1^ in Swedish schoolchildren participating in the national survey 2014

	OR (95% CI)
Sex	
Boy	1
Girl	2.1 (1.7–2.5)
Grade	
5	1
7	2.1 (1.6–2.7)
9	2.0 (1.6–2.6)
SES	
Both parent non-manual worker	1
One parent non-manual worker	1.3 (1.0–1-6)
No parent non-manual worker	1.5 (1.2–1.9)
Place of birth	
Sweden	1
Other	1.3 (0.9–1.8)
Family structure	
Nuclear family	1
Other	1.4 (1.1–1.7)

^1^Odds ratios with 95% confidence intervals from a logistic regression model with sex, grade, SES, place of birth and family structure as independent variables

**Fig. 2:**
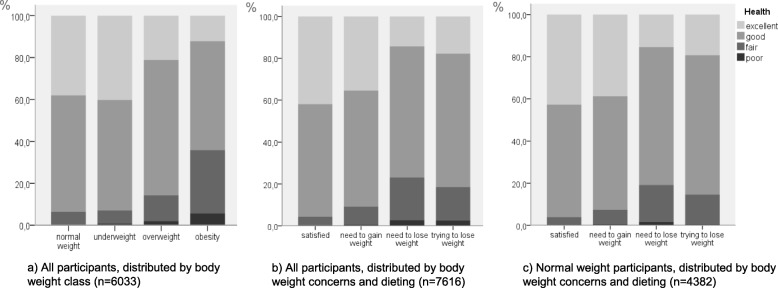
Self-rated health was related to body weight class (**a**), and to body weight satisfaction/dissatisfaction and dieting (**b**). The latter relationship remained when underweight, overweight and obese participants were excluded (**c**)

Dieting and body weight dissatisfaction were associated with perceived poor health (Fig. 2b, and Table 5, first panel). Participants who were dieting rated their health poorer than those who were satisfied with their weight. Dieters showed odds of reporting poor health that was four times higher than those who were satisfied with their weight. As body weight status was related to self-rated health (Fig. 2a) we adjusted for this in a second step, but dieting was still associated with poor self-rated health, OR 3.4, CI 2.6–4.4. This is illustrated in Fig. 2c, showing self-rated health in relation to dieting and body weight satisfaction/dissatisfaction in normal weight participants separately.

**Table 5:** Odds ratios and 95% confidence intervals of different aspects of health according to body weight dissatisfaction and dieting in Swedish schoolchildren participating in the national survey 2014

Health aspect	Prevalence (%)	*n*	Body weight concern and dieting			
			Satisfied	Need to gain weight	Need to lose weight	Trying to lose weight
Overall health						
Poor health^a^	9	7534	1	2.2 (1.7–2.9)	5.9 (4.8–7.3)	4.2 (3.4–5.2)
Poor health^b^		5950	1	2.1 (1.5–2.8)	4.9 (3.8–6.4)	3.4 (2.6–4.4)
Poor health^c^		4211	1	2.1 (1.4–3.3)	4.4 (3.2–6.1)	2.9 (2.0–4.0)
Not feeling well^a^	3	7351	1	2.3 (1.5–3.7)	3.5 (2.4–5.1)	3.8 (2.7–5.5)
Not feeling well^b^		5820	1	1.9 (1.1–3.3)	4.3 (2.7–6.9)	4.6 (3.0–7.3)
Not feeling well^c^		4158	1	2.0 (0.9–4.1)	3.3 (1.7–6.1)	3.2 (1.7–5.9)
Life satisfaction						
Low life satisfaction^a^	16	7432	1	1.9 (1.5–2.3)	2.7 (2.3–3.2)	3.2 (2.7–3.8)
Psychological						
Regularly feeling low^a^	6	7455	1	2.2 (1.6–3.0)	3.0 (2.3–3.8)	3.7 (2.9–4.7)
Regularly difficulties in sleeping^a^	13	7486	1	1.7 (1.4–2.1)	2.1 (1.7–2.5)	3.0 (2.5–3.6)
Physical						
Stomach-ache frequently^a^	4	7472	1	2.3 (1.6–3.3)	2.6 (1.9–3.5)	2.8 (2.0–3.8)
Headache frequently^a^	5	7491	1	1.5 (1.1–2.2)	2.3 (1.7–3.1)	2.8 (2.1–3.6)
Diagnosis						
Chronical disease^a^	21	7442	1	1.3 (1.1–1.5)	1.2 (1.0–1.4)	1.5 (1.3–1.8)
Social						
Bullied past months^a^	12	7556	1	1.9 (1.5–2.3)	1.8 (1.5–2.3)	2.6 (2.1–3.1)
Not accepted by teachers^a^	2	7572	1	0.9 (0.5–1.6)	1.9 (1.2–3.1)	3.2 (2.1–4.8)
Not accepted by classmates^a^	4	7487	1	1.6 (1.1–2.4)	2.4 (1.7–3.3)	3.2 (2.4–4.3)
Never dinner with parent^a^	2	7401	1	1.8 (1.1–3.0)	2.5 (1.6–3.9)	3.5 (2.3–5.3)
Never breakfast with parent^a^	14	7570	1	1.2 (1.0–1.5)	1.7 (1.5–2.1)	1.9 (1.6–2.3)
Can’t talk problems with family^a^	13	7375	1	1.2 (1.0–1.6)	1.6 (1.3–2.0)	2.1 (1.8–2.6)
Can’t talk problems with friends^a^	16	7349	1	1.3 (1.0–1.5)	1.5 (1.3–1.8)	1.7(1.4–2.0)
Behaviours						
Sugar sweetened beverages daily^a^	5	7542	1	1.8 (1.3–2.4)	1.6 (1.2–2.2)	1.4 (1.0–2.0)
Sweets daily^a^	4	7513	1	1.6 (1.2–2.3)	1.5 (1.0–2.0)	1.2 (0.8–1.7)
Not vegetables daily^a^	59	7512	1	1.2 (1.1–1.5)	1.5 (1.3–1.7)	1.0 (0.8–2.0)
Not fruit daily^a^	72	7591	1	1.1 (0.9–1.3)	1.4 (1.2–1.7)	0.9 (0.8–1.0)
Not breakfast every weekday^a^	27	7486	1	1.3 (1.1–1.5)	2.8 (2.4–3.2)	2.5 (2.2–3.0)
Not exercise weekly^a^	13	7301	1	1.7 (1.4–2.1)	2.1 (1.7–2.5)	0.7 (0.5–0.9)
TV ≥ 4 h/day weekdays^a^	21	7221	1	1.2 (1.0–1.5)	1.6 (1.4–1.9)	1.7 (1.4–2.0)
Smoking^a^	5	7621	1	1.5 (1.1–2.1)	1.9 (1.4–2.5)	3.2 (2.5–4.2)
Alcohol^a^	19	7563	1	1.2 (1.0–1.5)	1.5 (1.3–1.8)	2.2 (1.8–2.6)
Bed time after 24 weekdays^a^	6	7345	1	1.6 (1.2–2.1)	2.3 (1.8–3.0)	2.5 (2.0–3.3)

^a^Odds ratios with 95% confidence intervals from a logistic regression model adjusted for age and sex

^b^Adjusted for BMI class, age and sex

^c^Adjusted for SES, country of birth, family structure, BMI class, age and sex

Not only were attempts to lose weight related to reporting poor health and not feeling well. Participants who perceived a need to gain or lose weight were also more likely to report that they had a poor health and were not feeling well when compared with those satisfied with their weight. Moreover, Table 5 shows that body weight dissatisfaction was related to low life satisfaction and many different aspects of poor health. Dieting and body weight dissatisfaction were associated with self-reported chronical disease, psychological and somatic complaints, being bullied, not being accepted in school, seldom having family meals and not being able to talk about problems with family and friends.

Moreover, dieting was associated with behaviours as exercise, omitting breakfast and smoking, which the participants may regard as weight reduction strategies. Other behaviours like dedicating time for television viewing, going to bed late and consuming sugar sweetened beverages and alcohol were also associated with dieting.

Similar patterns of association were found for all six groups when stratifying the analyses in Table 5 by age and gender, although fewer associations were significant. When excluding participants without data on BMI in sensitivity analyses the results were not substantially different from those presented in Table 5 including all participants with data.

## Discussion

This paper adds to the present literature by showing that adolescent dieting is prevalent and increasing in Sweden, and that dieting and body dissatisfaction are related to perceived health status as well as various physical, psychological and social aspects of health.

In this national sample of Swedish 11- to 15-years olds dieting and body weight dissatisfaction was associated with perceived poor health and low life satisfaction. Dieting subjects were more likely to report poor health compared to those who were satisfied with their body weight. Furthermore, the ones who perceived a need to lose weight but did not put it into practice, were even more likely to rate their health as poor compared to those who were satisfied. These subjects, who wanted to reduce their weight, had odds to report poor health that was six times higher than those who reported body weight satisfaction. The majority of the Swedish schoolchildren reported a healthy weight, but the results show that also among those without overweight, attempts or perceived needs to reduce weight were present and associated with poor self-rated health. Similar findings have been found in other populations [27, 28]. Thus, it is important to recognize that adolescents of both sex and in all weight classes are concerned about weight, and to find out if they need different support to reach and maintain a healthy body image and weight.

### Dieting an increasing problem

According to the present study, dieting is increasing in Swedish schoolchildren of both sex. In the older age groups, the increase seems to be somewhat more pronounced in girls, and the prevalence was higher in girls already at the data collection 1993/94. However, the trends were significant for both girls and boys, and among the adolescents in grade five the trends were similar in boys and girls. Thus, even if the prevalence of dieting is lower in Sweden than in many other countries [29] it is an increasing health problem in both girls and boys.

### Associations with various psychological, physical and social health aspects

Dieting and weight dissatisfaction were not only associated with perceptions of poor health and illness in general, but also to different aspects of psychological, social and physical ill-health. This is in line with other studies showing associations between dieting or perceived need to lose weight and for example somatic [27, 28, 30] and psychological health complaints [27, 28], bullying [27], and family communication [14, 31]. However, in the present study we also demonstrated that perceived need to gain weight was associated with many of the examined health aspects. Thus, weight satisfaction was linked with self-reported psychological, physical and social health as well as overall health, life satisfaction and healthy behaviours. Even if the associations remained significant when adjusting for SES, family structure, foreign background, BMI class, age and sex it is impossible to state causality with the present study design. These associations could be an indication of causation in either direction or that family situation, parental involvement and other living conditions affect both body image and other health aspects.

Family meals, which might be a marker and facilitator of parental support and connectedness, is suggested to be a protective factor against eating disorders [32]. Previous studies have also demonstrated that eating with the family is associated with healthier diets, psychological wellbeing and decreased risk of obesity and unhealthy behaviours [33, 34].

### Associations with less healthy behaviours

The associations between dieting and health behaviours might just be a result of clustering behaviours [35]. Another explanation might be that smoking and omitting breakfast are strategies to lose weight [36]. Studies in other populations have also found dieting to be associated with unhealthy behaviours like smoking [37, 38]. Likewise, regular breakfast consumption has previously been shown to be less likely in overweight [39] and dieting [28, 38, 40] adolescents. Thus, omitting breakfast might be an attempt to lose weight and/or an unhealthy behaviour that result in weight gain. In any way, omitting meals should be considered in health promotion.

In the present study those being satisfied with their own body weight tended to report healthier food habits than the other participants. Dieting was not related to lower consumption of sweets and sugar sweetened beverages as shown in studies in other countries [28, 37]. Weight dissatisfaction was rather associated with higher consumption of these foods. Furthermore, the present study confirms that dieting adolescents do not consume fruit and vegetable more often compared to non-dieting adolescents who are satisfied with their body weight [28, 37]. Thus, dieting seems not to be the same as conforming to a dietary recommendations or dietary weight loss strategies recommended by health professionals.

### Limitations

One limitation of the present study is that adolescents can have different understanding of the word dieting which might influence how they respond on the question about weight reduction and body satisfaction. Another limitation is that body weight and height were self-reported and not measured. Self-report is likely lead to an underestimation of overweight and obesity prevalence [41, 42] and contribute to missing data for BMI. Due to a high proportion of missing data on height and weight, BMI could only be calculated for 78% of the participants. However, even if this study is based on self-reported data and that the participants are not followed longitudinally, the results are important for promotion of healthy habits as well as in prevention and management of obesity and eating disorders.

### Prevent dieting

Body dissatisfaction and disordered eating are delicate problems which must be tackled on many different levels [43], and awareness of dieting as a health problem is a good starting point. There is a risk that society in general and experts in particular may contribute to the unhealthy focus on body weight by highlighting the risk of obesity and the importance of overweight prevention. It is therefore encouraging to see an emerging salutogenic perspective in research with increased focus on body appreciation [16] and healthy attitudes towards eating [44].

In addition to aiming at healthy weight in young people, we should set the goal to prevent adolescents from dieting. To reach these goals it is important to always keep in mind to mediate and facilitate a balanced view of food habits, physical activity and body image, as well as to prevent unhealthy concerns and practices of weight control in itself. It is necessary to break the unhealthy spiral of weight gain, body weight focus and dieting.

Actions on many levels and arenas may promote a supportive youth environment with good role models and support in regular physical activity and balanced food habits for pleasure and health. Some examples on what to strive for are: schools that promote health literacy, healthy food habits and reflections about body ideals and norms and; sport clubs with sound policies, rules and information regarding weight reduction and dietary intake; social media without weight stigma, cyberbullying and unhealthy focus on appearance.

## Conclusions

The results indicate that adolescent dieting is prevalent and increasing among Swedish girls and boys. This is of great concern since previous studies have shown that dieting predicts eating disorders as well as obesity later on in life.

In the present study, dieters were more likely to perceive their health as poor than those satisfied with their weight. This also applied to those who wanted to lose or gain weight. In addition to these associations with overall health, dieting and body weight dissatisfaction were linked to unhealthy behaviours and multiple aspects of health. Thus, there is a need for action to prevent adolescent from dieting and rather empower them to body satisfaction and healthy food habits.

## Data Availability

The datasets generated and/or analysed during the current study are available in the HBSC Data Management Centre repository, https://www.uib.no/en/hbscdata/113290/open-access

## Abbreviations

ADHD: Attention deficit hyperactivity disorder
BMI: Body mass index
DVD: Digital versatile disc
h/day: Hour per day
HBSC: Health behaviour in school-aged children
OR: Odds ratio
SES: Socioeconomic status
TV: Television

## References

[1] Mendes V, Araujo J, Lopes C, Ramos E (2014). Determinants of weight loss dieting among adolescents: a longitudinal analysis. J Adolesc Health.

[2] Liechty JM, Lee MJ (2013). Longitudinal predictors of dieting and disordered eating among young adults in the U.S. Int J Eat Disord.

[3] Stice E, Mazotti L, Krebs M, Martin S (1998). Predictors of adolescent dieting behaviors: a longitudinal study. Psychol Addict Behav.

[4] Lowe MR, Doshi SD, Katterman SN, Feig EH (2013). Dieting and restrained eating as prospective predictors of weight gain. Front Psychol.

[5] Dulloo AG, Jacquet J, Montani JP, Schutz Y (2015). How dieting makes the lean fatter: from a perspective of body composition autoregulation through adipostats and proteinstats awaiting discovery. Obes Rev.

[6] Poobalan AS, Aucott LS, Precious E, Crombie IK, Smith WC (2010). Weight loss interventions in young people (18 to 25 year olds): a systematic review. Obes Rev.

[7] Muris P, Meesters C, van de Blom W, Mayer B (2005). Biological, psychological, and sociocultural correlates of body change strategies and eating problems in adolescent boys and girls. Eat Behav.

[8] Ricciardelli LA, McCabe MP (2003). Sociocultural and individual influences on muscle gain and weight loss strategies among adolescent boys and girls. Psychol Sch.

[9] Neumark-Sztainer D, Wall M, Larson NI, Eisenberg ME, Loth K (2011). Dieting and disordered eating behaviors from adolescence to young adulthood: findings from a 10-year longitudinal study. J Am Diet Assoc.

[10] Canadian Paediatric Society (2004). Dieting in adolescence, position statement. Paediatr Child Health.

[11] Neumark-Sztainer D, Paxton SJ, Hannan PJ, Haines J, Story M (2006). Does body satisfaction matter? Five-year longitudinal associations between body satisfaction and health behaviors in adolescent females and males. J Adolesc Health.

[12] Keel PK, Baxter MG, Heatherton TF, Joiner TE (2007). A 20-year longitudinal study of body weight, dieting, and eating disorder symptoms. J Abnorm Psychol.

[13] Ojala K, Vereecken C, Valimaa R, Currie C, Villberg J, Tynjala J (2007). Attempts to lose weight among overweight and non-overweight adolescents: a cross-national survey. Int J Behav Nutr Phys Act.

[14] Loth KA, MacLehose R, Bucchianeri M, Crow S, Neumark-Sztainer D (2014). Predictors of dieting and disordered eating behaviors from adolescence to young adulthood. J Adolesc Health.

[15] Whitehead R, Berg C, Cosma A, Gobina I, Keane E, Neville F (2017). Trends in adolescent overweight perception and its association with psychosomatic health 2002-2014: evidence from 33 countries. J Adolesc Health.

[16] Lemoine JE, Konradsen H, Lunde Jensen A, Roland-Levy C, Ny P, Khalaf A (2018). Factor structure and psychometric properties of the body appreciation Scale-2 among adolescents and young adults in Danish, Portuguese, and Swedish. Body Image.

[17] Meland E, Haugland S, Breidablik HJ (2007). Body image and perceived health in adolescence. Health Educ Res.

[18] El Ansari W, Clausen SV, Mabhala A, Stock C (2010). How do I look? Body image perceptions among university students from England and Denmark. Int J Environ Res Public Health.

[19] Borges A, Gaspar de Matos M, Diniz JA (2013). Body image and subjective well-being in Portuguese adolescents. Span J Psychol.

[20] ter Bogt TF, van Dorsselaer SA, Monshouwer K, Verdurmen JE, Engels RC, Vollebergh WA (2006). Body mass index and body weight perception as risk factors for internalizing and externalizing problem behavior among adolescents. J Adolesc Health.

[21] Brixval CS, Rayce SL, Rasmussen M, Holstein BE, Due P (2012). Overweight, body image and bullying--an epidemiological study of 11- to 15-years olds. Eur J Pub Health.

[22] Currie C, Nic Gabhainn S, Godeau E, International HNCC (2009). The health behaviour in school-aged children: WHO collaborative cross-national (HBSC) study: origins, concept, history and development 1982-2008. Int J Public Health..

[23] Fosse NE, Haas SA (2009). Validity and stability of self-reported health among adolescents in a longitudinal, nationally representative survey. Pediatrics..

[24] Cantril H (1965). The pattern of human concern.

[25] Cole TJ, Lobstein T (2012). Extended international (IOTF) body mass index cut-offs for thinness, overweight and obesity. Pediatr Obes.

[26] Bonthuis M, van Stralen KJ, Verrina E, Edefonti A, Molchanova EA, Hokken-Koelega AC (2012). Use of national and international growth charts for studying height in European children: development of up-to-date European height-for-age charts. PLoS One.

[27] Al Sabbah H, Vereecken C, Abdeen Z, Coats E, Maes L (2009). Associations of overweight and of weight dissatisfaction among Palestinian adolescents: findings from the national study of Palestinian schoolchildren (HBSC-WBG2004). J Hum Nutr Diet.

[28] Kelly C, Molcho M, Nic GS (2010). Patterns in weight reduction behaviour by weight status in schoolchildren. Public Health Nutr.

[29] Inchley J, Currie C, Young T, Samdal O, Torsheim T, Augustson L (2016). Growing up unequal: gender and socioeconomic differences in young people’s health and well-being.

[30] Fife B, Forste R. Physical and social factors associated with early adolescent headache and stomachache pain. Int J Adolesc Med Health. 2016. 10.1515/ijamh-2016-0062 27665418

[31] Al Sabbah H, Vereecken CA, Elgar FJ, Nansel T, Aasvee K, Abdeen Z (2009). Body weight dissatisfaction and communication with parents among adolescents in 24 countries: international cross-sectional survey. BMC Public Health.

[32] Langdon-Daly J, Serpell L (2017). Protective factors against disordered eating in family systems: a systematic review of research. J Eat Disord.

[33] Goldfarb S, Tarver WL, Sen B (2014). Family structure and risk behaviors: the role of the family meal in assessing likelihood of adolescent risk behaviors. Psychol Res Behav Manag.

[34] Martin-Biggers J, Spaccarotella K, Berhaupt-Glickstein A, Hongu N, Worobey J, Byrd-Bredbenner C (2014). Come and get it! A discussion of family mealtime literature and factors affecting obesity risk. Adv Nutr.

[35] Spring B, Moller AC, Coons MJ (2012). Multiple health behaviours: overview and implications. J Public Health (Oxf).

[36] Ojala K, Tynjala J, Valimaa R, Villberg J, Kannas L (2012). Overweight Adolescents' self-perceived weight and weight control behaviour: HBSC study in Finland 1994-2010. J Obes.

[37] Gabhainn SN, Nolan G, Kelleher C, Friel S (2002). Dieting patterns and related lifestyles of school-aged children in the Republic of Ireland. Public Health Nutr.

[38] Raffoul Amanda, Leatherdale Scott T., Kirkpatrick Sharon I. (2018). Weight Management, Weight Perceptions, and Health-Compromising Behaviours Among Adolescent Girls in the COMPASS Study. The Journal of Primary Prevention.

[39] Haug E, Rasmussen M, Samdal O, Iannotti R, Kelly C, Borraccino A (2009). Overweight in school-aged children and its relationship with demographic and lifestyle factors: results from the WHO-collaborative health behaviour in school-aged children (HBSC) study. Int J Public Health.

[40] Lattimore PJ, Halford JC (2003). Adolescence and the diet-dieting disparity: healthy food choice or risky health behaviour?. Br J Health Psychol.

[41] Ekstrom S, Kull I, Nilsson S, Bergstrom A (2015). Web-based self-reported height, weight, and body mass index among Swedish adolescents: a validation study. J Med Internet Res.

[42] Sherry B, Jefferds ME, Grummer-Strawn LM (2007). Accuracy of adolescent self-report of height and weight in assessing overweight status: a literature review. Arch Pediatr Adolesc Med.

[43] Ogden J, Murcott A, Belasco W, Jackson P, Mintz SW (2013). Eating disorders and obestity: symptoms of a modern world. The handbook of food research.

[44] Dockendorff SA, Petrie TA, Greenleaf CA, Martin S (2012). Intuitive eating scale: an examination among early adolescents. J Couns Psychol.

